# Characteristics of patients with thromboembolic disorders on warfarin therapy in resource limited settings

**DOI:** 10.1186/s12913-018-3537-4

**Published:** 2018-09-19

**Authors:** Zipporah Kamuren, Gabriel Kigen, Alfred Keter, Alice Maritim

**Affiliations:** 1 Department of Pharmacology and Toxicology, Moi University School of Medicine, P.O. Box 4606, Eldoret, 30100 Kenya; 2 Department of Pharmacology and Toxicology, Moi University School of Medicine, P.O. Box 4606, Eldoret, 30100 Kenya; 3 Academic Model Providing Access to Healthcare, P.O. Box 4606, Eldoret, 30100 Kenya; 4 Department of Pharmacology and Toxicology, Moi University School of Medicine, P.O. Box 4606, Eldoret, 30100 Kenya

**Keywords:** Warfarin, Interactions, INR stability, Factors, Outcomes

## Abstract

**Background:**

Warfarin is a drug with narrow therapeutic index used in the management of thromboembolic disorders. Several factors affect its plasma concentrations with a resultant risk of toxicity. We examined the database of patients on warfarin therapy in order to establish the factors that affect the stability of INR and correlated them to clinical outcomes in resource limited settings.

**Methods:**

We analysed retrospective data of patients admitted to adult medical wards at Moi Teaching and Referral Hospital (MTRH) in 2015. Inclusion criteria were patients with thromboembolic and related disorders and on warfarin treatment. Derived data included demographics, indications for warfarin use, co-prescribed drugs, co-morbidities, INR measurements, duration of hospital stay and clinical outcomes. Descriptive statistics were used to summarize the data. Pearson’s correlation coefficient was used to assess relationships between duration of hospitalization and number of INR tests. Regression splines were used to capture INR trends during the follow up period. Data was analysed using R v. 3.3.1.

**Results:**

A total of 310 patients had thromboembolic disorders, out of which 63 met the study criteria.

The median age was 48 years, while the mean number of INR measurements was once every four days. Majority of patients did not achieve stable INR values, with only two having consecutive INR values within therapeutic goal. Patients who died had high INR levels. The median duration of hospital stay was 9 days (IQR: 7.0, 16.5). There was a significant correlation between length of stay in hospital and the number of times that INR were measured (Corr = 0.667, *p* < 0.001). The two most common indications for warfarin were DVT (64.4%) and atrial fibrillation (24.7%). All the patients had one or more comorbid conditions except for 11 with DVT alone, with cardiovascular diseases and infections being the most frequent, and on concomitant medications, majority of which are known to interact with warfarin.

**Conclusions:**

It was difficult to achieve stable INR under the prevailing conditions despite the frequent tests.

The potential factors that may have contributed to the fluctuations include drug-drug interactions, frequency of INR tests, comorbidities and the short duration of hospital stay.

## Background

Warfarin is an oral anticoagulant that acts by inhibiting of vitamin K-dependent clotting factors (II, VII, IX, X) and anticoagulant factors, proteins C and S [64, 67]. The drug is useful in the management of various thromboembolic disorders including venous thromboembolism (VTE), atrial fibrillation (Afib), valvular heart disease and post-myocardial infarction [64]. Its main advantages include availability in oral formulation, efficacy and low cost, thus making it an important drug especially in resource limited settings [62, 71]. However, it has several limitations, the most significant being its narrow therapeutic window. It causes bleeding at high concentrations but is ineffective at low dosage. The variable dose response necessitates frequent monitoring of its plasma levels [8, 28]. In addition, it is usually co-administered with several other drugs thus predisposing it to several drug-drug interactions [67]. Warfarin also interacts with food and herbs which are widely used in African countries [10, 18, 31]. Such interactions may either result in reduced efficacy or toxicity [10, 35, 64, 66]. The novel oral anticoagulants (NOACs) such as dabigatran, rivaroxaban, apixaban and edoxaban have several practical advantages compared to vitamin K antagonists (VKAs) such as warfarin [55]. These include rapid onset/offset of action, predictable anticoagulant response and lower potential for food and drug interactions. They are however still currently expensive and not easily accessible in developing countries. In addition, VKAs have a broader spectrum of indications, standardized monitoring tests and established reversal strategies compared to the NOACs [55, 61]. Warfarin therefore still remains the most widely used oral anticoagulant in the management of conditions that require long term anticoagulation in these settings [49].

The efficacy of warfarin is monitored by measuring prothrombin time (PT), usually converted to International Normalized Ratio (INR). The desired INR range varies according to disease, but in most cases between 2 to 3. Risk of bleeding increases proportionately with INR values above 4 [46]. INR stability is therefore crucial for optimization of warfarin therapy in the management of thromboembolic disorders. Regular monitoring is thus required in the adjustment of warfarin dose in order to maintain INR levels within the desired range [28]. Since most patients who require warfarin are likely to be on other medications, mainly due to comorbid conditions, the risk of interactions with the drug is high [17, 42]. A study conducted in USA reported that 81.6% of patients on warfarin and co-administered drugs received at least one potentially interacting drug [30, 70]. A related study established that about 74% of patients on warfarin were on concurrent treatment with drugs that interact with warfarin, with 13% being contraindicated [54]. Comorbid conditions and adherence to medication do also influence the INR stability. Additionally, the cost and accessibility of the concomitant drugs is also a major determinant, especially in resource-limited settings [4, 15, 56].

The main aim of the study was to investigate the characteristics of patients on warfarin therapy admitted to the adult medical wards at MTRH, with a view to optimization of warfarin therapy. Factors that affect stability of INR were evaluated and correlated with the clinical outcomes. These included the presenting thromboembolic disorder, comorbidities, co-administered drugs, frequency and values of INR measurements and duration of stay at the hospital. The potential for interactions between concomitant drugs with warfarin, and clinical outcomes were also assessed (based on literature review) and recorded.

## Methods

Permission to conduct the research was obtained from the Institutional Research and Ethics Committee (IREC) of Moi University College of Health Sciences and MTRH [Approval Number: FAN; IREC 1559; 2016] [25]. A retrospective descriptive study of patients on warfarin therapy in adult medical wards at MTRH in 2015 was carried out. The hospital is based in the North-rift region of Kenya and is a referral hospital for patients from the larger Western region of Kenya [40]. The hospital has partially digitalized its records and employs the World Health Organization (WHO) International Classification of Diseases-10 (ICD-10) system to categorize diseases [24]. This system was utilized to facilitate selection of the appropriate patient files. Inclusion criteria were patients with or at risk of thromboembolic disorders and on warfarin treatment. Patients with thromboembolic and related disorders but not on warfarin were excluded. Disease conditions known to require the use of warfarin were entered into the database which subsequently generated identification file numbers (computer-generated) for patients diagnosed with the diseases. The search was limited to adult medical wards for the year 2015. Physical files were then retrieved from the records office and files that met the inclusion criteria were selected. Misclassified files, those with incomplete records or absent at the records office during the time of data collection were excluded. Key disease conditions included VTE, Afib, rheumatic heart disease, stroke, heart failure, myocardial infarction, heart valve replacement, gangrene, aortic stenosis and superior vena cava syndrome. The desired data was extracted from the selected patient files using a predetermined working sheet. These data included patient demographics, indications for warfarin use, INR values, number of times that INR was measured and warfarin dosages throughout the hospital stay. Also included were comorbid conditions, concomitant drugs, records of adverse events, duration of stay in hospital, outcome at the end of admission and any other relevant information. Drugs.com website, an on-line drug interactions checker website, was used to identify concomitant drugs that interact with warfarin. Based on this information, we categorized the drug interactions as being either major, moderate or minor; including their effects on INR levels. Relevant published articles were also utilized [10, 14, 17, 37, 42].

### Data management

De-identified data was entered into a Microsoft excel spreadsheet. These included age, sex, indication for warfarin use, dose range, adverse effects, number of days spent in hospital and outcomes. Comorbid conditions were classified as per the WHO ICD-10 category. INR variables derived included the number of times it was carried out, range and values from the onset of warfarin therapy. In addition, the number of times when INR values were less than 2 (< 2), between 2 to 3, 4 to 8, and greater than 8 (> 8) during therapy were recorded. The identified interactions were categorized into major, moderate or minor interactions and effects on INR were reported as either an increase or decrease in values. Co-administration was defined as presence of at least a one-day overlap between the intake of warfarin and interacting drug [54].

### Statistical analysis

Categorical variables such as sex, co-morbid conditions and outcomes (mortality, discharge or transfers) among others were summarized using frequencies and the corresponding percentages. Continuous variables such as age, INR levels, and duration of stay in hospital among others were summarized using mean and the corresponding standard deviation (SD) whenever the Gaussian assumptions were holding; otherwise median and the corresponding inter quartile range (IQR) was used. Gaussian assumptions were assessed using Shapiro-Wilks test and histograms. Correlation between the number of INR measurements and duration of hospital stay was assessed using Pearson’s correlation coefficient. We reported the associated estimate and the *p*-value. We used generalized estimation equations with cubic regression splines to model the overall and outcome specific trends of INR levels over the duration of hospitalization. Data analysis was performed using R: A language and environment for statistical computing [51].

## Results

A total of 6819 patients were admitted to the adult medical wards during the study period (2015) as per the information from Health Records and Information Services Office of MTRH. There were 310 computer-generated patient identification file numbers (patients with thromboembolic disorders), out of which 63 met the study criteria (on warfarin) with 36 (57.1%) being females. The median age of the patients was 48 years (IQR: 31.5, 68.5) with a range of 15 to 95 years. All the patients had one or more comorbid conditions except for 11 with DVT as the single recorded condition. Sixty-one patients were on management for active thromboembolic disorders, whereas two were on prophylaxis for DVT. Of the two, one was a 91-year-old with a history of ischaemic stroke, while the other had TB adenitis secondary to HIV/ADS, and at risk of DVT. Table 1 outlines the indications of warfarin use and the comorbid conditions. The two most common indications for warfarin were deep venous thrombosis (DVT) (65.1%) and atrial fibrillation (Afib) (22.2%). Of the comorbid conditions, cardiovascular diseases were the most frequent (52.4%), followed by infections (44.4%) and gastrointestinal disorders (GIT) (15.9%) respectively. Among the infections, eight patients (12.7%) were HIV positive and 5 (7.9%) had tuberculosis (TB).

**Table 1:** Indications for warfarin use and comorbid conditions (*N* = 63)

Variable	n (%)
Indication for warfarin use	
DVT	41 (65.1%)
Afib	14 (22.2%)
Cardiac thrombus	4 (6.5%)
PE	7 (11.1%)
Prophylaxis for DVT	2 (3.2%)
Internal jugular vein thrombosis	1 (1.6%)
Comorbidities	
Cardiovascular related conditions	33 (52.4%)
Infections	
HIV	8 (12.7%)
TB	5 (7.9%)
Other infections	15 (23.8%)
GIT disorders	10 (15.9%)
Neoplasms	8 (12.7%)
Blood disorders	7 (11.1%)
Respiratory related conditions	6 (9.5%)
Endocrine, nutritional and metabolic disorders	6 (9.5%)
Genitourinary tract disorders	5 (7.9%)
Mental and behavioural disorders	5 (7.9%)
Post-pregnancy related conditions	2 (3.2%)
Skin and subcutaneous tissue diseases	3 (4.8%)
Musculoskeletal disorders	2 (3.2%)
Nervous system disorders	1 (1.6%)

### Duration of stay in hospital and clinical outcomes

Out of the 63 patients, one patient was admitted three times while six were admitted twice, within that year, giving a total of 71 admissions (Table 2). The median duration of stay in hospital was 9 days (IQR: 7.0, 16.5), with a minimum of 3 and maximum of 104 respectively. Forty-two (66.7%) patients were discharged home and 3 (4.8%) were transferred to other units within the hospital or other hospitals. Bleeding episodes were noted in 4 patients, though it was not clear from the records whether they were linked to warfarin. This included epistaxis while in hospital (at a warfarin dose of 3 mg/day); upper GIT bleeding (as indication for admission) in a patient who had been on warfarin at home; haemoptysis in a patient on warfarin but admitted for treatment of lobar pneumonia, and several episodes of nose bleeding in a patient later diagnosed with acute myeloid leukaemia. In all the four cases, warfarin was stopped and the INR values monitored.

**Table 2:** Number of admissions and duration of stay in hospital (*N* = 63)

Variable	n (%) or Median (IQR)
Number of admissions within the year	
One	56 (88.9%)
Two	6 (9.5%)
Three	1 (1.6%)
Duration of hospital stay (Days)	9 (7.0, 16.5)
Range (Min. – Max.)	1–78
Outcome	
Transferred	3 (4.8%)
Discharged Home	42 (66.7%)
Died	18 (28.6%)

The mortality report is summarized in Table 3. A total of 18 deaths (28.6%) were recorded, with the highest occurring amongst patients with Afib or DVT. The two patients with DVT died. The rest are as shown in the table.

**Table 3:** Mortality amongst patients on warfarin therapy (*N* = 18)

Indication for warfarin use	Total no. of deaths [n (%)]
Afib	5 (27.8%)
Cardiac thrombus	1 (5.6%)
DVT	5 (27.8%)
DVT + PE	2 (11.1%)
PE	3 (16.6%)
Prophylaxis	2 (11.1%)

### Correlation between INR tests, trends and clinical outcomes

**Fig. 1:**
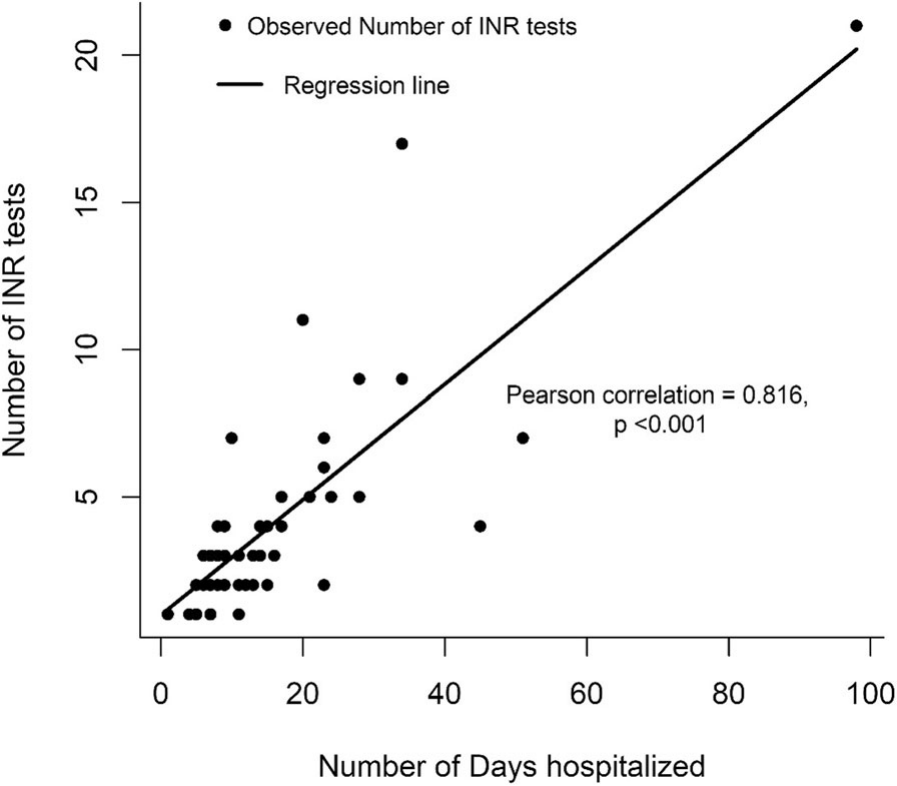
Correlation of INR testing and the number of days spent in hospital

**Fig. 2:**
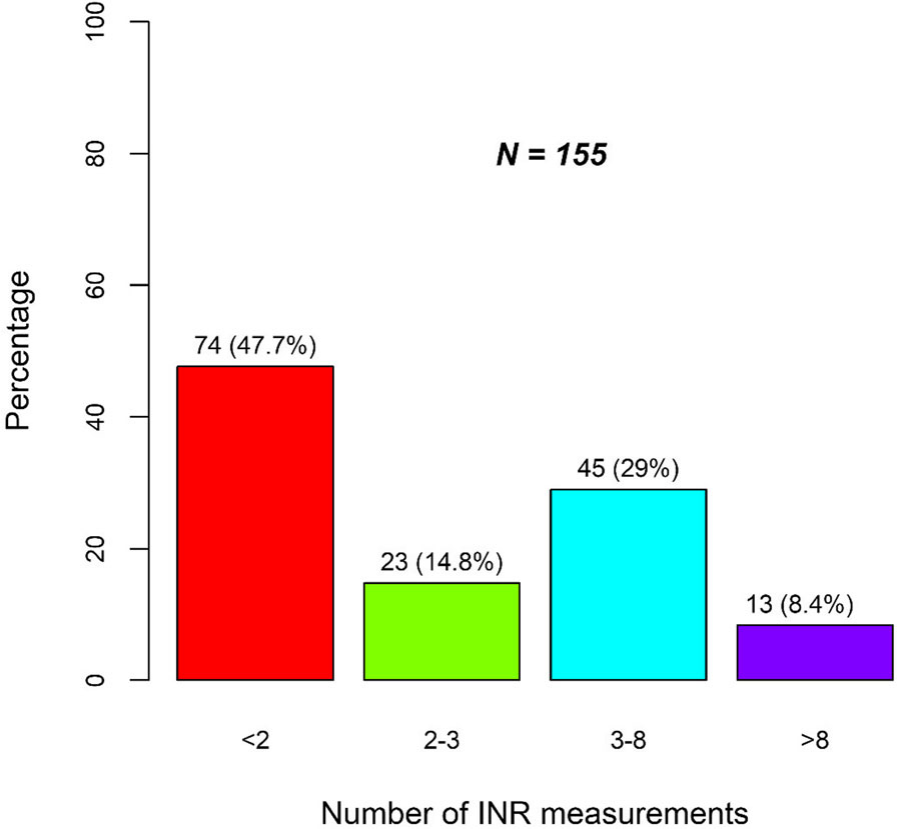
INR values after the initiation of warfarin therapy

The summary of the results for INR tests are as illustrated in Fig. 1 and Table 4. INR were measured for a median of 3 times (IQR: 2.0, 4.0) over a range of 1 to 21, translating to about once every 3 to 4 days which is within the recommended outpatient range. There was a significant correlation between the length of stay in hospital and the number of times that INR were measured (Corr = 0.816, *p* < 0.001) **[**Fig. 1**].** INR was not performed on 5 patients during admission as they were either already on warfarin or had a very short duration of stay in hospital. A total of 155 INR measurements were performed whilst they were on warfarin therapy in the wards (Fig. 2). Twenty-three (14.8%) of the values were within the desired range (2–3), 74(47.7%) were less than 2; while 45(29%) were between 3 and 8. Thirteen (8.4%) of the 155 INR values from 7 (12%) patients were beyond 8. Six of the 7 patients with at least one INR > 8, died. Only two patients (9.5%) had consecutive INR test values within the therapeutic goal of 2 to 3, with one having three and the other five. Warfarin was administered at doses ranging between 1 to 8 mg per day except for 2 patients whose doses was increased over time to13 and 20 mg per day respectively in order to achieve an INR of between 2 to 3.

**Table 4:** Relationship between the number of INR measurements per patient and outcomes

Outcome levels	Number of patients (N)	Number of INR measurements		^k^*P*-value
		Median (IQR)	Min. – Max.	
Death	18	3.5 (2.2, 6.5)	1.0–21.0	
Discharged Home	38	2.5 (2.0, 3.0)	1.0–17.0	0.058
Transferred to another Hospital	2	5.0 (5.0, 5.0)	5.0–5.0	

^k^Kruskal Wallis test

**Fig. 3:**
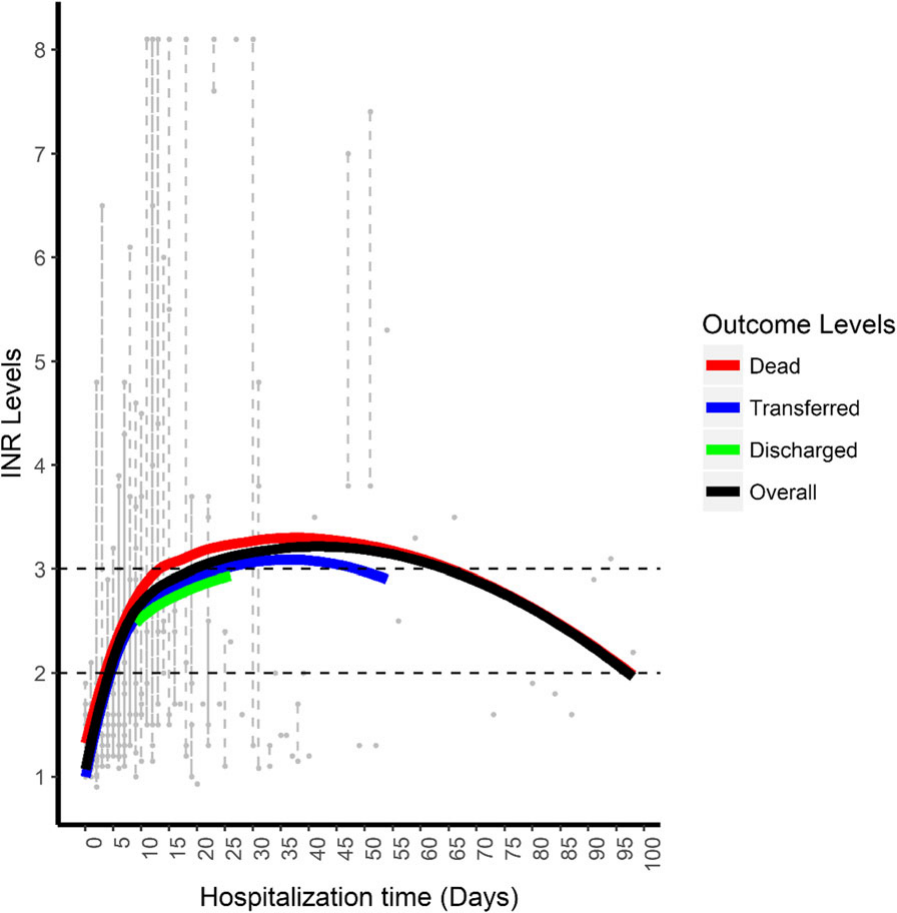
Overall and outcome stratified trends of INR levels over the duration of stay in hospital

Majority of the patients did not achieve stable INR values. The overall and individual trends of INR levels over the duration of stay in hospital are as illustrated in Fig. 3. The patients began with low INR levels and which increased over the first 15 days before attaining an asymptote where they began to decline steadily. Some patients showed a decline that goes below the initial level at admission. The outcome stratified trends of INR levels over the duration of stay in the hospital were also modelled. Patients who died demonstrated higher levels of INR throughout the hospitalization period, and the patients who were transferred to other facilities had lower INR levels. There is no evidence of the patients consistently having their INR levels within the recommended range of 2–3 (the gap between the dotted lines) during the days of hospitalization.

The relationship between the number of INR measurements and outcomes is presented in Table 4. Kruskal-Wallis test, was used to compare the median. The median number of INR measurements for the patients who died was higher than among those who were discharged, and those transferred to other facilities (3.5 (IQR: 2.2, 6.5) vs. 2.5 (IQR: 2.0, 3.0) vs. 5.0 (5.0, 5.0). There was, however, no sufficient evidence from the data to explain the difference in INR measurement among the outcome levels (*p* = 0.058). A comparison of the patients who died to those who were either discharged home or transferred to another facility did not reveal any evidence of differences in number of INR measurements (3.5 (IQR: 2.2, 6.5) vs. 3.3 (IQR: 2.0, 4.0), *p* = 0.096).

### Co-administered drugs

**Fig. 4:**
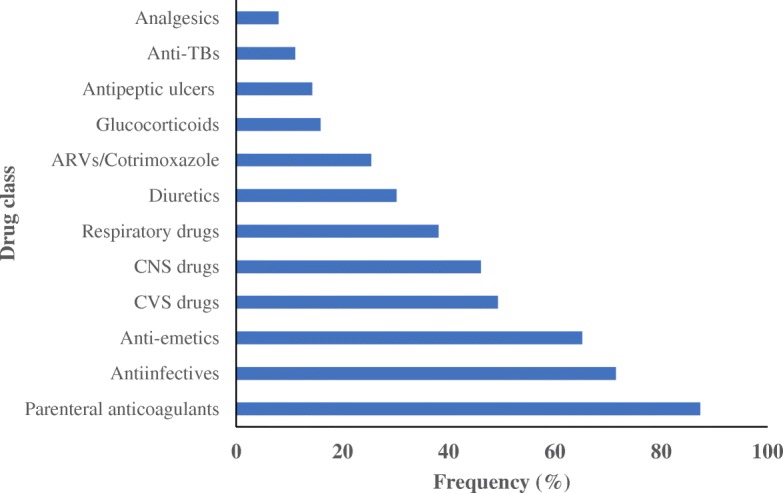
Classes of drugs used by the patients

Out of the 63 patients, 55 had been started on either heparin or enoxaparin at the initiation of warfarin therapy. All the patients were on other drugs alongside warfarin. The average number of drugs per admission was 7.5 with a range of 1 to 18. Figure 4 shows the distribution of the co-administered drug as per their respective classes. Anti-infective agents and analgesics were the most co-prescribed classes of drugs. In many cases, drugs were substituted with similar ones in case they were not available. Most of the anti-infective agents are known to have significant interaction with warfarin, including ceftriaxone, metronidazole, co-trimoxazole, fluconazole and rifampicin. Table 5 outlines a summary of the concomitant drugs used by the patients, and potential for their interaction with warfarin based on literature search from the drug interaction websites [10, 14, 17, 37, 42]. The drugs with major warfarin-related interactions included metronidazole 16(25%) patients, cotrimoxazole 9(14%), rifampicin 5(8%) and diclofenac 5(8%) amongst others. Drugs with moderate interaction with warfarin (and high usage) included ceftriaxone 34(54%) patients, esomeprazole 26(41%) and tramadol 21(33%).

**Table 5:** List of prescribed drugs known to interact with warfarin, level of interaction and frequency of use

Drug Class	Drug	Frequency (No/%)	Degree of interaction	Effect on INR
Anti-infective Agents	Metronidazole	16 (25)	Major	↑
	TMP/SMX	9 (14)	Major	↑
	Fluconazole	4 (6)	Major	↑
	Levofloxacin	3 (5)	Major	↑
	Ciprofloxacin	1 (2)	Major	↑
	Clarithromycin	1 (2)	Major	↑
	Ceftriaxone	34 (54)	Moderate	↑
	Amoxicillin/clavulanate	10 (16)	Moderate	↑
	Azithromycin	9 (14)	Moderate	↑
	Doxycycline	7 (11)	Moderate	↑
ARVs	Efavirenz	3 (5)	Moderate	↑/↓
	Nevirapine	1 (2)	Moderate	↓
Anti-TBs	Isoniazid	5 (8)	Moderate	↑
	Rifampicin	5 (8)	Major	↓
Anti-ulcer drugs	Esomeprazole	26 (41)	Moderate	↑
	Omeprazole	7 (11)	Moderate	↑
Analgesics	Diclofenac	5 (8)	Major	–
	Tramadol	21 (33)	Moderate	↑
	Paracetamol	20 (32)	Moderate?	↑
	Celecoxib	1 (2)	Moderate	–
CNS drugs	Carbamazepine	2 (3)	Moderate	↓
	Phenytoin	4 (6)	Moderate	↑/↓
	Fluoxetine	2 (3)	Moderate	↑
Glucocorticoids	Prednisone	4 (6)	Moderate	↑/↓
	Dexamethasone	1 (2)	Moderate	↑/↓
	Hydrocortisone	1 (2)	Moderate	↑/↓
Diuretics	Furosemide	26 (41)	Minor	↑
	Spironolactone	12 (19)	Minor	↓
CVS drugs	Amiodarone	16 (25)	Major	↑

Key

↑ Increased INR

↓ Decreased INR

Amoxiclav Amoxicillin-clavulanic acid combination

SMZ/TMP Trimethoprim/sulfamethoxazole combination

ARVs Antiretroviral agents

## Discussion

The study investigated the characteristics of patients admitted to the adult medical wards with thromboembolic disorders and on warfarin treatment, in order to establish the factors that affect INR stability with a view to optimization of warfarin therapy in resource limited settings. INR, a measure of warfarin activity, is affected by several factors and its stability is crucial for optimization of the drug’s therapy. Although it was difficult to analyse the results owing to the high number of co-morbidities, high number of co-administered drugs and quality of the data; we established several factors that may have affected the INR stability. This included comorbidities, drug-drug interactions, frequency INR measurements and duration of stay at the hospital. Frequent change in co-administered drugs may have also contributed to the fluctuation in INR values.

Maintaining INR within therapeutic range is still a universal challenge. Previous studies conducted under strictly-administered adherence protocols have only yielded time-in-therapeutic ranges of between 50 and 70% [47, 50, 53]. The introduction of computer-assisted dosing of warfarin in developed economies has led to improved outcomes [52]. Keen and frequent monitoring of INR is however vital for optimal warfarin use in order to prevent bleeding and thrombotic events. Previously, MRTH employed a traditional laboratory method of INR measurement; but now utilizes the finger-stick testing using a point-of-care device [48]. The devise is convenient to use with immediate results thus allowing for faster therapeutic decision-making, which in turn boosts safer use of warfarin. Because of the availability of a point-of-care testing and pharmacy staff dedicated to the anticoagulation clinic at MTRH, the average frequency of INR testing is once every 4 days [48]. The results from our study indicate that INR tests were performed for a median of 3 times over a range of 1 to 21 days, translating to about once every 3 to 4 days. However, most patients did not attain steady INR values while in hospital, with only two patients (9.5%) having consecutive INR test values within the therapeutic goal of 2 to 3. The frequency of INR measurements may have contributed to this instability, as the intervals may have been inadequate. Ideally, INR levels should be measured daily at the initiation of warfarin therapy until the target value is achieved, and maintained for at least two consecutive days. Moreover, any changes in co-prescribed drugs and presence of comorbid conditions may require more frequent monitoring [26, 38, 43, 44]. The duration of hospital stay was also a contributory factor to the vacillating INR values, as it correlated to the number of times that the tests were performed. Due to bed-space constrains and resource limitations, many patients were discharged once stable, but before attaining a steady INR within the therapeutic range; to be followed up as outpatients in the pharmacy-run anticoagulation clinic and other relevant clinics. From our study, only 23 (14.8%) of the 155 INR tests conducted while the patients were in hospital were within the desired range. The patients started with low INR levels which increased within the first 15 days before attaining an asymptote and declining steadily to below the initial level at admission in some patients.

Drug-drug interactions were also identified as a potential contributor to the unstable INR values. We identified several drugs that were routinely used on the patients which interact significantly with warfarin. Metronidazole, cotrimoxazole, rifampicin and diclofenac were classified from the literature as causing major interactions that result in increased INR values [5, 14]. Ceftriaxone, although classified as having moderate interactions was one of the most widely used drugs, in 54% of the patients [11, 14, 58]. Rifampicin increases the metabolism of warfarin thus decreasing the INR. Tramadol and paracetamol were the commonly prescribed analgesics, and have been shown to have a moderate interaction with warfarin [14, 16]. The frequent changes of the drugs prescribed to patients on a day-to day basis may have also exacerbated the situation. We identified several instances whereby there were frequent changes of the co-prescribed drugs such as antibiotics. Like many other resource restrained countries, the availability of a specific drug on a continuous basis is still a major challenge in Kenya [21, 32, 69]. In addition, some disease conditions have been found to affect warfarin’s blood anticoagulant effect were present in some patients in this study group, including renal and hepatic dysfunction [9]. The liver is the principle organ for the synthesis of clotting factors and metabolism of many drugs including warfarin; and if dysfunction may result in warfarin toxicity [20, 41]. Moreover, renal failure [34, 57], thyroid disease [23] and malignancies [45] can all complicate anticoagulation by increasing INR and bleeding tendency. Other factors that may have contributed to the unstable INR include food intake [12], failure to adhere to instructions (skipping doses or overdosing), lack of timely follow-up and dose adjustment. Polymorphism of the enzymes involved in warfarin metabolism also affect warfarin sensitivity. Most Africans are known to carry the typical CYP 2C9 allele with some diversity in the VKORC1 genes [39].

In our study, majority (56%) of the patients were less than 54 years old; although the distribution of the patients on warfarin treatment cut across all age groups. This differs from high income countries whereby anticoagulation is more commonly used in the elderly population [3, 29, 68]. VTE was the commonest indication for warfarin use (64.2%); followed by Afib (21.9%). This also contrasts with several outpatient studies conducted in developed countries which have reported Afib as being the most frequent indication for warfarin therapy followed by VTE [2, 29, 43, 68]. The risk factors for VTE in our study included stroke secondary to hypertension, congestive heart failure, malignancy and chronic infections. The high burden of HIV, a risk factor for VTE and its associated comorbid conditions such as TB, contribute not only to increased numbers of VTE, but also for its appearance in the younger age group [7, 13, 65]. DVT was associated with one or more comorbid conditions in all but 11 patients (17.2%), thus necessitating the use of warfarin along with other medications. This is evinced by the number of drugs that a patient was on during a single admission (average of 7.5, range 1–17) thus increasing the chances that the drugs will interact with warfarin and complicate its therapy. RHD was a common condition in our study (7 patients, 10.9%), all being managed for Afib; at an age range of 15 to 48 years. Being a young person’s disease, RHD is known to be more common in Africa than developed countries which may explain the high incidence references [63].

The major complication associated with warfarin therapy is bleeding [22, 33]. Bleeding risk increases with an INR value greater than 4, age (over 65 yrs), malignancy, renal insufficiency and liver failure [1, 6, 19, 22, 27, 33, 36, 59]. Haemoptysis can be due to a variety of clinical conditions including TB, suppurative pneumonia, acute bronchitis, and lung cancer [60]. Although patients in this cohort who presented with bleeding had underlying conditions that are known to cause bleeding, it is plausible that warfarin may have likely played a role in the appearance and intensity of the bleeding.

## Conclusions

It was difficult to achieve stable INR under the prevailing conditions despite the frequent tests, and the out of range values may have significantly impacted on the clinical outcomes. The potential factors that may have contributed to the fluctuations include drug-drug interactions, number and frequency of INR tests, comorbidities, frequent changes in co-administered drugs and the short duration of hospital stay. Daily monitoring of INR levels at the initiation of warfarin therapy until target values are achieved and maintained for at least two days may improve the treatment outcomes. Continuous availability of co-administered drugs, such as antibiotics during the dosage range is also crucial in order to minimize drug-drug interactions. An improvement in patient records including follow up is also important. Ingenious/cheap methods including the use of colour-coded pages dedicated for information on anticoagulation will simplify follow up as the anticoagulation entries will not be mixed up with other patient notes.

### Study limitations

The retrospective nature of the study coupled with the imperfect record keeping meant that the study could not be as rich as intended. The small sample size, which could have been limited by the search terms and the fact that not all computer-identified files were present in the storage area at the time of data collection, means the results may not be extrapolated to the population at large. Additionally, there were many and varied coexisting comorbid conditions and administered drugs that affect INR. It was therefore difficult to untangle the effect of each comorbidity on INR values. There were several other confounding factors including the short duration of hospital stay coupled with the small sample size which may have been inadequate to provide enough data on the INR trends. Moreover, the retrospective nature of the study and inadequate entries did not allow for detection of the actual impacts of the drug interactions.

## Abbreviations

Afib: Atrial fibrillation
CYP: Cytochrome
FFP: Fresh Frozen Plasma
GIT: Gastrointestinal Tract
HIV: Human Immunodeficiency Virus
ICD: International Classification of Diseases
INR: International Normalized Ratio
IREC: Institutional Research and Ethics Committee
MTRH: Moi Teaching and Referral Hospital
P-450 DVT: Deep Vein Thrombosis
PE: Pulmonary embolism
PT: Prothrombin Time
RHD: Rheumatic Heart Disease
TB: Tuberculosis
USA: United States of America
VKORC1: Vitamin K epoxide reductase complex subunit 1
VTE: Venous Thromboembolism
WHO: World Health Organization
